# Impacts of Mesopredator Control on Conservation of Mesopredators and Their Prey

**DOI:** 10.1371/journal.pone.0137169

**Published:** 2015-09-11

**Authors:** L. Mike Conner, Gail Morris

**Affiliations:** Joseph W. Jones Ecological Research Center, Newton, GA, United States of America; University of Queensland, AUSTRALIA

## Abstract

Declining large carnivore populations, increased habitat fragmentation, declining interests in fur trapping, and other anthropogenic factors can all lead to increased mesopredator populations and these may negatively impact biodiversity. Lethal mesopredator control potentially mitigates some of these effects but can be controversial, largely because impacts on mesopredator populations have not been evaluated. Estimating these impacts may reduce controversies while increasing our understanding of when lethal control may be beneficial. Therefore, we analyzed published mesopredator removal data to determine if mesopredator removal rates changed over time. Removals of medium,(e.g., raccoons (*Procyon lotor*) or red foxes (*Vulpes vulpes*), and large, i.e., bobcats (*Lynx rufus*) or coyotes (*Canis latrans*), mesopredators were consistent from year to year and over the duration of study (i.e., number removed during the first and last years of studies were similar). In contrast, removals of small mesopredators, e.g., weasels (*Mustela* spp.) or spotted skunks (*Spilogale putorius*), declined over the duration of study. Study area size, number of species targeted for removal, and duration of removal effort were poor predictors of removal rates. Our analyses suggest that: (1) control, as typically implemented, is unlikely to cause negative long-term impacts on populations of medium and large mesopredators but may negatively impact small mesopredators, (2) if mesopredator control benefits prey, recurring removals will generally be needed to maintain benefits, and (3) timing of removals will be important to achieve management goals. We suggest that mesopredator control efforts are frequently spatially structured harvests from continuously distributed populations. This may explain (1) why removal of small mesopredators declined over time; whereas, medium and large mesopredator removals remained consistent, and (2) why some prey failed to respond to mesopredator control efforts.

## Introduction

Many of the world’s large carnivore populations are declining because of land use change and human conflict [1]. The loss of large carnivores has cascading effects throughout ecosystems, and these cascading effects often include mesopredator (herein, a mammalian predator species with an adult body mass < 34 kg and typically 13–16 kg; [2]) release and subsequent declines in mesopredator prey populations [3]. Additionally, some mesopredator populations thrive in human-modified landscapes, creating human-wildlife conflicts [4]. Not surprisingly, increased mesopredator abundance and associated prey declines are frequently used as rationale for mesopredator control [5]. However, varied public attitudes toward lethal control often provide a cloak of controversy around predator removals [6].

Consensus among scientists can profoundly affect public opinion of controversial issues [7], but that consensus is lacking with regard to predator control (see [5], [8], [9] for examples). Effects of predator, primarily mesopredator, control efforts on their prey populations have been well studied (see reviews [5], [10–13]). In contrast, there has been no summary of the impacts of mesopredator removal on mesopredator populations. In the absence of such data, consensus among scientists regarding the long-term impacts of mesopredator control efforts on mesopredator populations is unlikely.

Some mesopredator removal studies have documented long-term reductions in mesopredator abundance as a result of lethal control efforts. However, these declines were generally accompanied by other decimating factors (e.g., habitat loss and fragmentation; [14]), or occurred in isolated patches [15] where immigration was hindered. In other cases, historically strong fur markets encouraged trapping over large areas and may have suppressed mesopredator populations [16]. In contrast, predator control to enhance prey populations generally takes place on individual parcels of land surrounded by what are essentially predator refuges. For example, over 90% of studies reviewed in Salo et al. [5] took place on areas < 100 km^2^, and studies that took place at larger spatial scales were generally associated with control of large carnivores as opposed to mesopredators. It is unclear if control efforts at these smaller and more common spatial scales are more or less likely to have lasting impacts on mesopredator populations.

The ability of mesopredator populations to respond rapidly to lethal control via immigration [17] may help explain results of mesopredator removal studies that failed to benefit prey. This is fairly common, as 24% of predator removal studies had no effect on prey populations [5]. Assuming effective removal techniques are available and applied with sufficient intensity, immigration of predators from surrounding areas may reduce efficacy of predator control [17]. Understanding how local populations of mesopredators respond to removal efforts will provide better information to conservation biologists and wildlife managers regarding timing of mesopredator removal efforts and potential need for continuous or recurring mesopredator control efforts.

To better understand the impact of mesopredator control efforts on mesopredator populations, we analyzed published multiyear mesopredator removal studies to determine: (1) variation in number of mesopredators removed from year to year and over the duration of study, and (2) whether this variation was affected by mesopredator body size, study area size, number of species targeted for removal, and duration of removal (i.e., number of years removal occurred). We restricted our analyses to mesopredators because they commonly experience a positive numerical response to the loss of top carnivores [3], [18] and anthropogenic activities [4], and control of mesopredators may help to mitigate some of the undesirable effects of trophic cascades (e.g., altered behavior or reduced abundance of small prey [19], [20]), mesopredators are often the foci of predator control efforts, and experiments that manipulate mesopredator abundance are relatively common [5]. Finally, we used results from our analyses to provide recommendations for management and future research.

## Methods

### Data collection

We retrieved studies cited in [5] and conducted a literature search of Web of Science and Biosis databases for papers with combinations of the following terms in the titles: (predator OR predation) AND (control, effect, experiment, impact, manipulation, removal, OR reduction). Our search of databases concluded on 25 June 2014.

Following the search of databases, we filtered publications based on title and eliminated studies that obviously did not manipulate terrestrial mesopredator populations (e.g., marine systems, invertebrate studies, simulations, etc.). We evaluated remaining publications to ensure that mesopredator manipulation was accomplished by removal or translocation, as opposed to mesopredator addition or exclusion. Finally, to be included in our analyses, studies must have removed mesopredators for > 1 year and recorded number of mesopredators removed or catch-per-unit-effort (CPUE) for each removal period.

If researchers removed predators from multiple sites and provided site-specific data, we treated sites independently in our analyses. For each study site, we recorded the number of mesopredator species targeted for removal and the number of animals removed each year to the lowest taxonomic level reported. We also recorded study area size, duration of study, and whether or not predator population sizes were assessed independently (e.g., some authors reported an index of mesopredator populations relative to removal efforts).

### Data Analyses

To determine if mesopredator removals varied from one year to the next, we calculated a ratio of removals using the equation for annual finite rate of population growth (λ) using (λ = *N*_*t+1*_ */N*_*t*_) described in (pages 93–94 in [21]) where N_*t*_ = number of removals at time *t* and N_t+1_ = number of removals at time *t* + 1. We then log-transformed the data to center the data around zero and reduce skew; thus, ln(λ) < 0.0 indicates removals declined between years, ln(λ) = 0.0 indicates consistent removals between years, and ln(λ) > 0.0 indicates removals increased from one year to the next. Similarly, we calculated a statistic associated with the duration of each study (λ_*d*_) using (λ_d_ = *N*_*end*_ /*N*_0_) where *N*_0_ = removals during the first removal effort of the study and *N*_*end*_ = removals during the last removal effort of the study. We log-transformed λ_*d*_ and considered ln(λ_*d*_) representative of the cumulative effects of mesopredator removals over the entire study. In some cases, studies reported CPUE instead of number of animals removed; when this occurred, we used CPUE in lieu of number of animals removed.

Log-ratios, i.e., ln(λ) or ln(λ_*d*_), were calculated for each species when data were available. We also partitioned mesopredators into three body size classes: (1)small, with adult mass typically ≤ 2.0 kg; i.e., American mink (*Neovision vison*), weasels, stoats (*Mustela* spp), pine martens (*Martes martes*), eastern spotted skunks (*Spilogale putorius*), yellow mongoose (*Cynictis penicillata*), cape grey mongoose (*Herpestes pulverulentus*), striped polecat (*Ictonyx striatus*), blackfooted cat (*Felis nigripes*), common gennet (*Genetta genetta*), and suricate (*Suricata suricatta*); (2) medium, with adult mass typically 2.0–10 kg; i.e., foxes, raccoons (*Procyon lotor*), striped skunks (*Mephitis mephitis*), American badgers (*Taxidea taxus*), Virginia opossums (*Didelphis virginiana*), nine-banded armadillos (*Dasypus novemcinctus*), and raccoon dogs (*Nyctereutes procyonoides*); and (3) large, with adult mass typically > 10kg; i.e., bobcats (*Lynx rufus*) and coyotes (*Canis latrans*), and calculated log-ratios based on class. Finally, because some control efforts removed multiple species but only reported overall removals, we combined all mesopredators for each study site and calculated ln(λ) and ln(λ_*d*_) associated with overall removals. We considered the log-ratios as our response variables for all further analyses.

To facilitate analyses, we partitioned data into 6 data sets based on the response variable: (1) ln(λ) calculated for each species (or species group as we combined members of the genera *Mustela* and *Neovison* into a weasel/mink group and members of the genera *Vulpes* and *Urocyon* into a fox group) and then averaged (arithmetic mean) across years for each site, (2) ln(λ_*d*_) calculated for each species or species group for each site, (3) ln(λ) calculated for each mesopredator size class and then averaged across years for each site, (4) ln(λ_*d*_) calculated for each mesopredator size class for each site, (5) ln(λ) calculated for all mesopredator removals and then averaged across years for each site, and (6) ln(λ_*d*_) calculated for all removals for each site. We did not treat these data as repeated measures because some study sites (n = 18) only provided one ln(λ) estimate for analyses, i.e, removals were not carried about for > 2 years, and would have required deletion of too many observations. By averaging over time, we treated multiple estimates of ln(λ) within a removal site as sub-samples; thus, the removal site served as the experimental unit in all analyses. Averaging was not required to use removal site as the experimental unit when considering ln(λ_*d*_) as the response variable because this statistic was calculated over the duration of study. For example, if removal of a species occurred on a given study site for 5 years, there would be 4 estimates of ln(λ) for that species, and the average of these would have been used in species-specific analyses. However, there would have only been one estimate of ln(λ_*d*_) based on the first and last removals.

We calculated summary statistics for ln(λ) and ln(λ_*d*_) for each mesopredator species or species group, mespredator size class, and for the total number of mesopredators removed. We graphed means and 90% CIs for all estimates of ln(λ) and ln(λ_*d*_) and used a one sample t-test with alpha = 0.10 to evaluate whether summary statistics differed from zero, indicating that removal rates changed over time.

We then used a general linear modeling approach to develop models predicting average ln(λ) or ln(λ_*d*_) for each of the 6 datasets described above. For the species-specific models, our predictor variables included species removed (SPECIES), number of species removed during the study (NUMSPP), and study area size (AREA, in ha) to predict ln(λ); we added duration of study (DURATION, in years) when predicting ln(λ_*d*_). Similarly, for the body size class models, we used size class of mesopredator (CLASS; small, medium, or large, treated as an monotonic ordinal variable), NUMSPP, and AREA to predict average ln(λ), adding DURATION to predict ln(λ_*d*_). Finally, for the total removals model, we used NUMSPP, and AREA to predict average ln(λ) and added DURATION when predicting ln(λ_*d*_).

Because there were relatively few predictor variables available for model development and because we were interested in the importance of individual predictors, we developed a single additive model predicting average ln(λ) and ln(λ_*d*_) for each modeling effort. We evaluated the significance of parameter estimates (i.e., parameter estimates were considered significant if they differed from zero using an alpha level of 0.10) to determine whether or not a particular parameter was informative (i.e., an important predictor). All models were developed with the glm function in R version 3.1.3 statistical software [22] using a Gaussian distribution and identity link.

## Results

Our literature search provided 2,862 potential publications. We eliminated all but 416 based on title. Of these, only 51 citations representing 34 studies met our criteria for analyses (S1 Table). Further examination of these 34 studies indicated that they provided 66 independent study sites. Multiple removal sites within a study were common, with half of studies reporting data from ≥ 2 study sites. Average study site size was 28995 ha (median 5950 ha; range = 50–772900 ha). Number of years that mesopredators were removed averaged 4.3 (median = 3; range = 2–58). Number of species targeted for removal averaged 3.12 (median = 2; range = 1–10).

### Species-specific analyses

**Fig 1:**
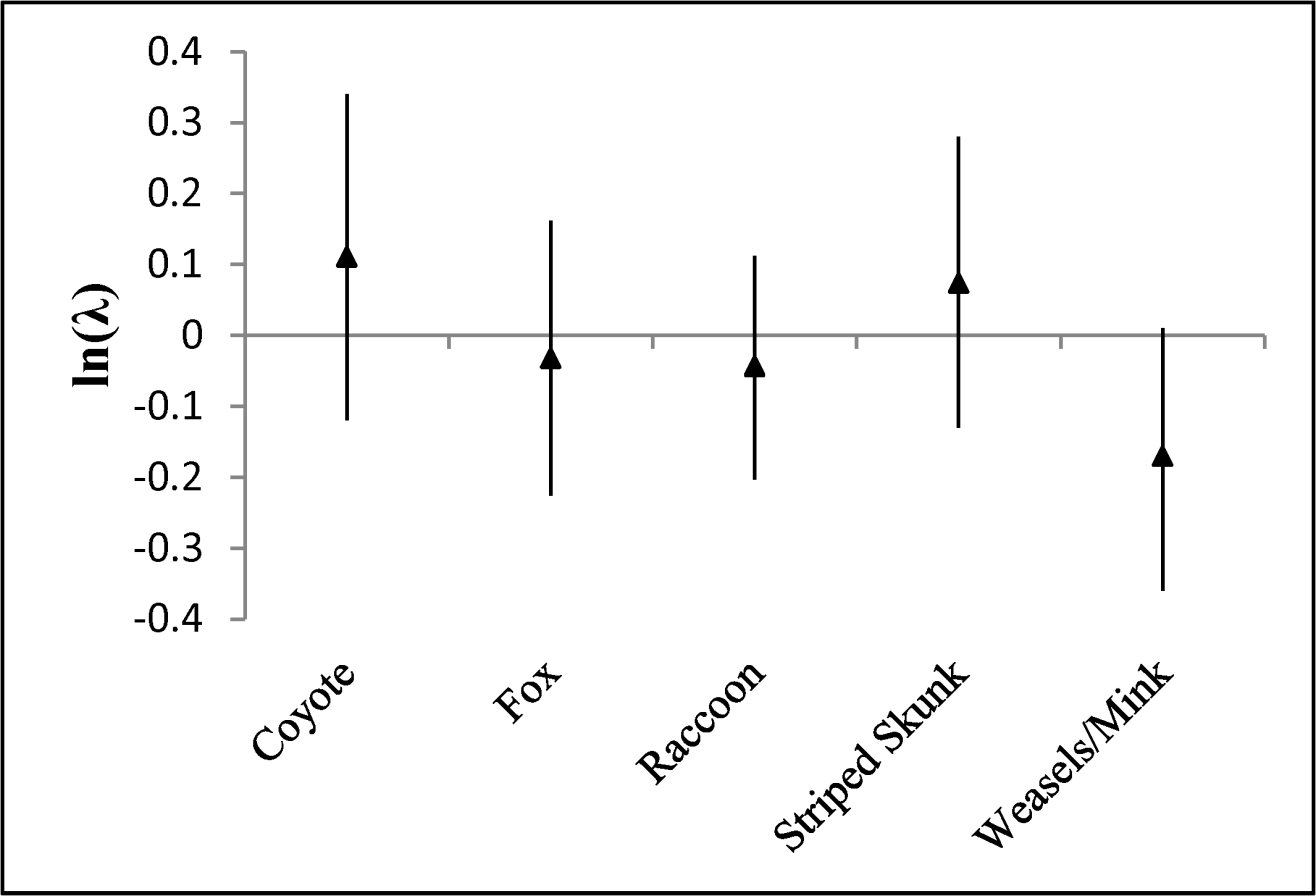
Mean (triangles) and 90% confidence intervals for ln(λ) = ln(number of animals removed during a removal period/number of animals removed during the prior removal period). An ln(λ) = 0 indicates removal rates did not vary from year to year. Data were obtained from published predator control studies.

There were sufficient data (≥ 10 removal sites) to perform species-specific or species-group analyses for 5 mesopredators: coyotes, foxes (predominantly *Vulpes vulpes* but 3 studies also reported *Urocyon cinereoargenteus*), raccoons, weasels/mink (*Mustela* and *Neovison* spp), and striped skunks. Average ln(λ) did not differ for any species or species group examined, suggesting that removals were relatively constant from one year to the next (Fig 1). Our modeling of average ln(λ) indicated that there were no informative parameter estimates (P > 0.10, all cases).

**Fig 2:**
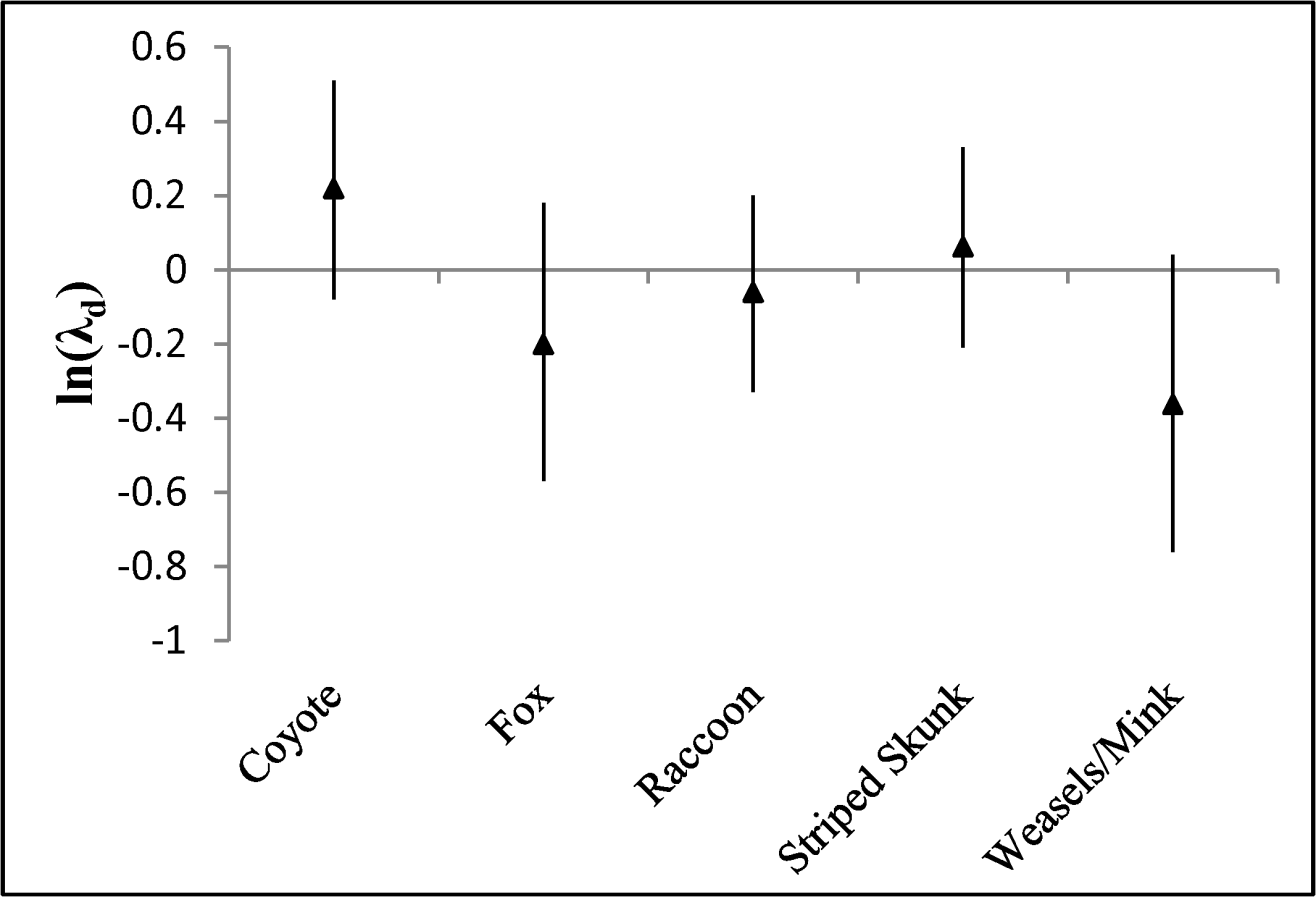
Mean (triangles) and 90% confidence intervals for ln(λ_d_) = ln(number of animals removed during the initial removal period/number of animals removed during the last removal period). An ln(λ_d_) = 0 indicates removal rates did not vary over the course of study. Data were obtained from published predator control studies.

When we analyzed species-specific removal data associated with the duration of study, results differed slightly. As with ln(λ), none of the ln(λ_d_) differed from zero (Fig 2). However, the parameter estimate associated with the weasel/mink species-group (beta = -0.54; P = 0.058) suggested that weasel/mink removals declined over the duration of study. Parameter estimates for all other species and species groups, AREA, NUMSPP, and DURATION were not informative.

### Size class analyses

**Fig 3:**
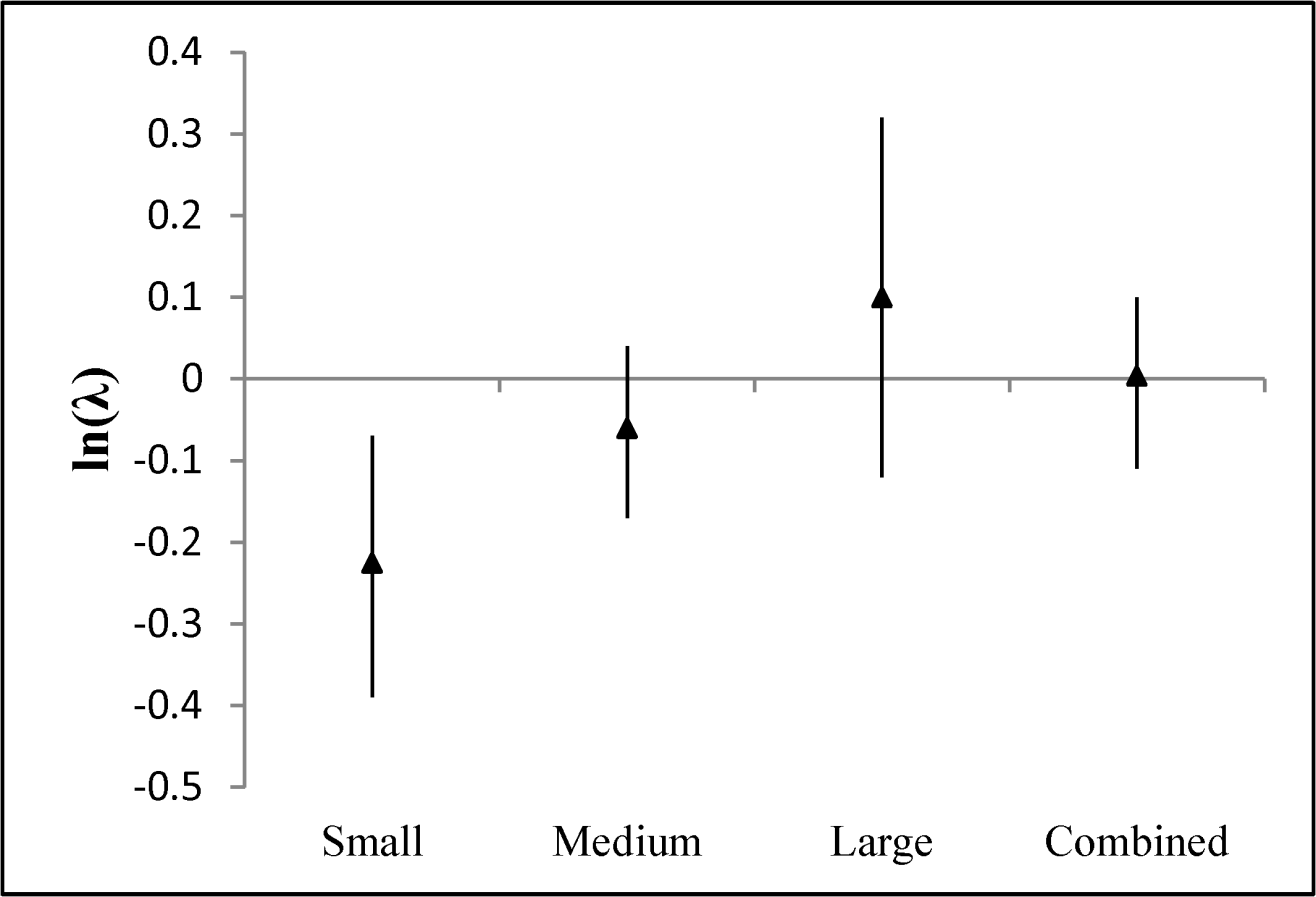
Mean (triangles) and 90% confidence intervals for ln(λ) = ln(number of animals removed during a removal period/number of animals removed during the prior removal period). Small = mesopredators with average mass < 2 kg, Medium = mesopredators with body mass typically 2–10 kg, Large = mesopredators with body mass typically > 10 kg, Combined = all mesopredators considered together. An ln(λ) = 0 indicates removal rates did not vary from year to year. Data were obtained from published predator control studies.

Average ln(λ) associated with small mesopredators was < 0.0 (t_26_ = -2.44, P = 0.02), suggesting that removals of small mesopredators tended to decline between consecutive removals; this was not observed for medium and large mesopredators (Fig 3). Results from the linear model showed the parameter estimate for CLASS as a predictor of average ln(λ) differed (beta = 0.16, P = 0.03) from zero suggesting that average ln(λ) increased as mesopredator size class increased. Remaining parameter estimates were non-informative.

**Fig 4:**
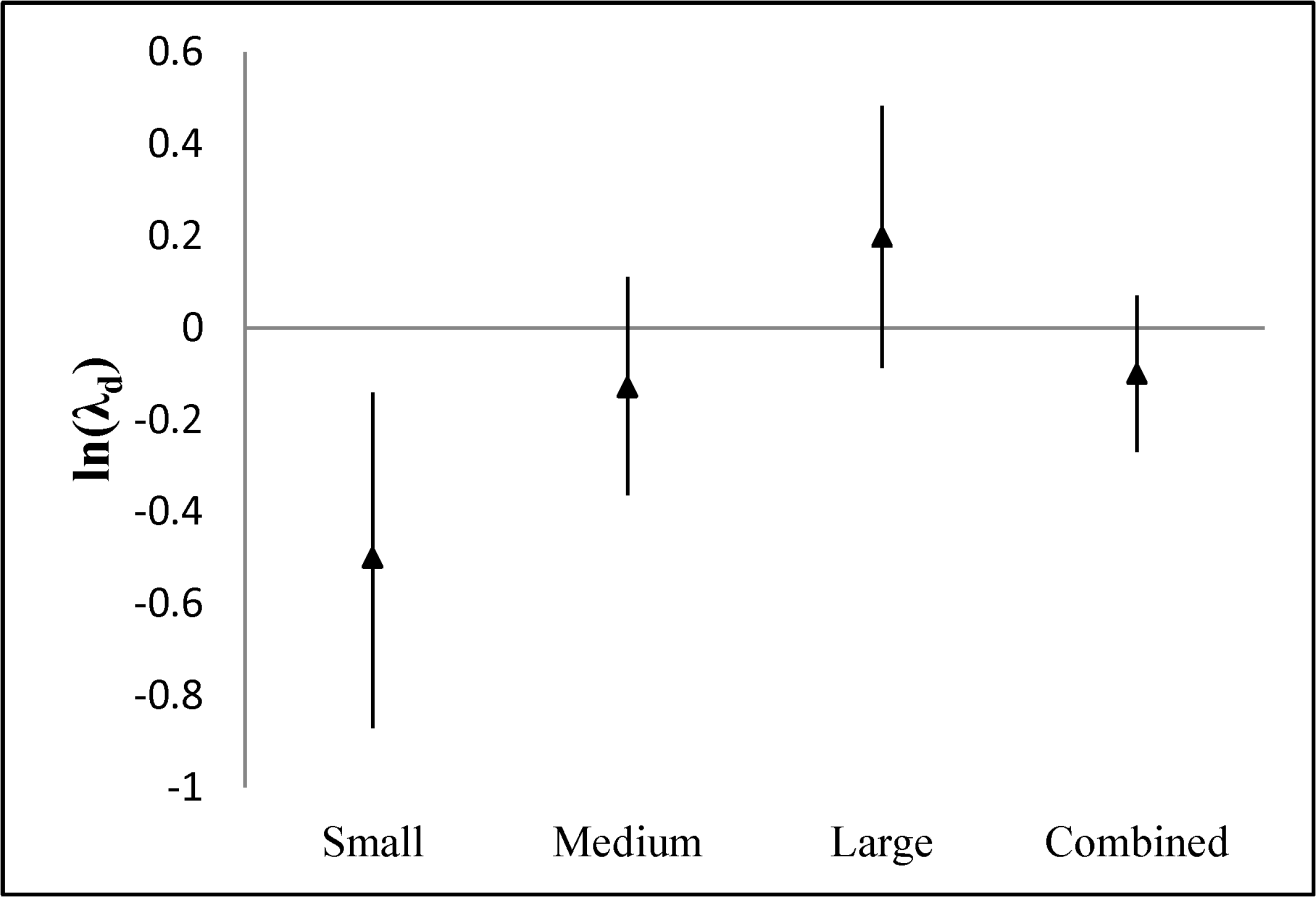
Mean (triangles) and 90% confidence intervals for ln(λ_d_) = ln(number of animals removed during the initial removal period/number of animals removed during the last removal period). Small = mesopredators with average mass < 2 kg, Medium = mesopredators with body mass typically 2–10 kg, Large = mesopredators with body mass typically > 10 kg, Combined = all mesopredators considered together. An ln(λ_d_) = 0 indicates removal rates did not vary over the course of study. Data were obtained from published predator control studies.

Small mesopredator ln(λ_d_) was also < 0.0 (t_25_ = -2.39, P = 0.02), suggesting that small mesopredator removals declined between the first and last removal periods; whereas, removals of medium and large mesopredators remained similar between the first and last removal efforts (Fig 4). The parameter estimate for CLASS as a predictor of ln(λ_d_) (beta = 0.36, P = 0.007) indicated that ln(λ_d_) increased as mesopredator size class increased. Remaining parameter estimates were non-informative.

### All mesopredators combined

When all mesopredators were combined, average ln(λ) did not differ (t_65_ = −0.06, P = 0.95) from zero (Fig 3). The model predicting average ln(λ) from NUMSPP and AREA suggested that neither parameter was informative. Similarly, ln(λ_d_) did not differ (t_65_ = − 0.95, P = 0.34) from zero (Fig 4) and modeling efforts to predict ln(λ_d_) from NUMSPP, AREA, and DURATION revealed no informative parameters.

## Discussion

Whenever conclusions are drawn from published literature, publication bias has the potential to profoundly affect those conclusions, particularly in meta-analyses [23]. Tests for publication bias have been developed to determine how many missing studies might exist and the effect that these studies might have had on the outcome of a meta-analysis [24], [25]. These tests are based on evaluating the relationship between effect size and sample size of studies used in the meta-analysis, for example the ‘trim and fill’ method [24]. However, data used in our work were presented as ancillary data and were not analyzed beyond simple reporting of the number of animals removed. This is evident when considering the number of potential papers resulting from our literature search relative to the number of studies with data that were suitable for inclusion in our study (n = 34). Because these data were not statistically analyzed in the context they were originally reported, there were no effect sizes associated with these data; thus, publication bias was not a consideration in our synthesis.

For our analyses to represent more than a simple statistics exercise, it is important that a meaningful relationship exists between changes in mesopredator abundance and our response variables, ln(λ) and ln(λ_d_). We suggest this was the case. Harvest records have been used for decades as indices of abundance [26], [27]. Incorporating removal effort can improve reliability of abundance indices that are derived from harvests [28]. Unfortunately, most predator control studies did not report removal effort; thus, it was not possible to evaluate effects of effort in our analyses. However, because removals were most often conducted to evaluate effects of predator removal on prey populations, we expect removal efforts were intensive, as ensuring adequate removal was necessary for a meaningful treatment to exist. In other words, researchers should have implemented intensive removal efforts to provide the best opportunity for evaluating prey response. For example, Kilgo et al. [29] estimated that annual removals of coyotes actually exceeded their abundance prior to initiation of removal, providing evidence that any prey response, or lack thereof, was real and not the result of insufficient removal efforts. Further, because mesopredator removal numbers were obtained from published studies, we suggest that it was reasonable to assume that removal efforts within a given study were held relatively constant as part of research protocol. Our analyses support this assumption. If increased removal effort occurred over time and if these removals resulted in declining mesopredator numbers, we would expect a negative relationship between duration of study (DURATION) and ln(λ_d_), and this was not observed in any of our models.

Our modeling of species-specific ln(λ_d_) suggested that the weasel/mink species-group experienced decreased removals over the duration of study. In contrast, there was no evidence that coyotes, striped skunks, foxes, or raccoons experienced similar declines. When mesopredators were partitioned into size classes, small mesopredator captures declined both between years and over the duration of study, and models suggested a positive association between mesopredator size and both response variables. Because the small size class was largely comprised of weasels and mink it is helpful to consider this size class and species grouping collectively.

Population recovery following harvest can only occur through some combination of reproduction and immigration. In the weasels and mink, litter sizes can be quite large with average litter sizes among species ranging from 4–12 and reported densities ranging from 10–120/km^2^ [30]. Dispersal distances among mammals are strongly influenced by body size and home range size [31]; thus, small mesopredators would be expected to have the smallest dispersal distances of the 3 size classes examined in our study. Studies reporting removals of small mesopredators took place on sites with a median size of 11.85 km^2^. If we assume only 10 small mesopredators / km^2^, expected abundance of these mesopredators on a median-sized study area would be approximately 119 animals. In contrast, densities of some of the larger mesopredators are generally much lower; for example, coyote densities average 0.2–0.4/km^2^ [32] and striped skunk densities average 1.8–4.8/km^2^ [33]. Considering an overall median study area size of approximately 60 km^2^, the potential proportion of the overall population impacted by removal would be greater for small mesopredators than for medium or large mesopredators (i.e., greater numbers of small mesopredators were exposed to removal efforts simply because they are small and have small home ranges). Thus, large removal areas relative to dispersal distances of small mesopredators and greater proportion of the population being exposed to removal efforts may explain why removals of small mesopredators declined over time while removals of larger mesopredator did not.

McCullough [34] suggested that populations can be harvested to provide a sustained yield by ensuring harvests are spatially structured such that both harvest and refuge areas exist across the landscape in which the harvested species occurs. Under this model, harvest occurs on only a portion of the available landscape. By varying the amount of the landscape subjected to harvest, a maximum sustained yield can be obtained. There is no need for quotas or bag limits to ensure a sustained yield under this model; other than being restricted to certain areas, there are no restrictions on harvest. This approach to managing harvests is not widely utilized by game managers, perhaps because harvest quotas are easier to implement. However, the goal of mesopredator removal, though often not stated as such, is to locally eradicate or minimally provide a substantial reduction in a mesopredator population. Thus, operational predator control efforts fit this conceptual model because they entail intensive removal efforts and typically take place on discrete parcels set within a larger landscape where mesopredators are essentially unharvested. We hypothesize that McCullough’s [34] harvest model explains the relatively constant mesopredator removals we observed with regard to medium and large mesopredators as well as the tendency for removals of small mesopredators to have longer-lasting effects. We suggest that body size and home range-related influences on dispersal distances of small mesopredators relative to large mesopredators [31] explains the longer-lasting effects of removal on small mesopredators.

Other than variables related to type of mesopredator removed, such as species group or body size, none of the examined predictors of ln(λ) or ln(λ_d_) were informative. We found this lack of informative predictors interesting. Perhaps the most interesting non-informative variable was AREA. Lack of a study area size effect on our response variables at first seems contradictory to the hypothesis that most predator removals are well represented by McCullough’s [34] spatially structured harvest model. It seems we should have observed a negative relationship between AREA and our response variables; this was not the case. We interpret the non-informative nature of AREA to be indicative that areas of study sites were below the sustained-yield threshold. Also interesting was the lack of DURATION as a predictor of ln(λ_d_), suggesting that the observed decline in removal rates associated with small mesopredators primarily occurred early in the study. That NUMSPP had no effect on any of our analyses suggests removal efforts that are focused on one or two species have no greater impact on those species than studies that take the more general approach of controlling a suite of mesopredator species.

The relatively consistent year-to-year removal rates of medium and large mesopredators has implication for conserving prey species. Consistent removals from one year to the next emphasize that time required for local populations of mesopredators to reach pre-removal levels should be considered when implementing a mesopredator control plan. Our analyses indicate that recovery occurs within a year for medium and large mesopredators, but because most studies reported removals on an annual basis, we were unable to robustly address time required for mesopredator populations to recover following control efforts. However, limited evidence suggests that immigration of large mesopredators can rapidly recover their populations following lethal control. For example, Henke and Bryant [35] removed coyotes seasonally for 8 consecutive seasons from 5,000 ha study sites. Using removal data from this study, we calculated an average seasonal ln(λ) = 0.02 ± 0.31, indicating that coyote immigration was sufficient to restore the study area population in only 3–4 months. In another study, VanGilder et al. [36] removed 22 coyotes and 10 bobcats from an 800 ha study area during a 6-month period. Here, number of removals may be reflective of extreme mesopredator densities, but we suggest that immigration during the removal period provides a better explanation for the number of removals from such a small area.

Although our analyses suggested that the individual species examined here responded to harvest similarly, variation among species may exist at finer temporal scales than we were able to assess (i.e., some species immigrate faster than other). Additionally, immigration following removal would likely be influenced by factors such as habitat quality [37] and mesopredator density in the surrounding area [38].

Approximately half (n = 16) of the studies meeting criteria for our analysis provided an independent index of predator abundance (S1 Table). Effects of mesopredator removal on these indices were inconsistent among studies. Predator indices were not impacted by removal efforts in some studies [39–41]. In other studies, population indices declined as a result of the removal effort, but recovered soon after [42–46]. Other studies had mixed results, with indices of predator abundance showing declines in some sites or years of the study but increases in other sites or years [47], [48]. Still others indicated population indices declined and remained suppressed during the duration of study [29], [35], [49], [50].

Interestingly, all of the studies reporting declining indices provided explanations that remain consistent with our synthesis. Cypher and Scrinver [49] indicated that coyote indices were declining prior to implementation of removal, providing evidence that other factors were at play. Coyote removals in the Henke and Bryant [35] study resulted in a 50% reduction in predator density, but this reduction was measured before and immediately following approximately 2 years of seasonal removal efforts; thus, immigration did not have time to occur following the last removal effort. Similarly, Kilgo et al. [29] reported a 78% decline in coyote scat collection each year of a 3 year removal effort relative to the number of scats collected prior to initiating coyote removal. In this study, collections were only performed in May following cessation of removal efforts in April. These latter studies indicated that (1) researchers were able to substantially reduce predator populations during a period of interest, (2) These reductions did not result in declines in captures over time, and (3) immigration of mesopredators from surrounding areas best explains consistency of captures over time. The Kirkwood [50] study is the only study to document a population that was apparently declining solely as a result of mesopredator removal efforts. This study reported on 58 years of red fox removal efforts. Early efforts at removal were accomplished using a bounty system which was replaced by more organized control efforts that spanned 25 years. Complete eradication was the goal during the last 5 years of the study. This study was conducted on an island with little connectivity to the mainland, meaning the red fox population was relatively isolated. Thus, we would predict that this population could experience declines due to control as predicted by McCullough’s [34] model. This apparently has occurred as the authors concluded that red foxes were nearing eradication from the area. Collectively, reported population indices were in agreement with our observation that removals of medium and large mesopredators remained relatively stable from year to year.

Because removal of medium and large mesopredators generally results in only short-term reductions in predation risk, timing of removal is critical and will need to be recurrent, or in some cases continuously implemented, to initiate and maintain prey response. Whether removals should be continuous or recurrent largely depends on the temporal vulnerability of the prey. If a prey species is susceptible to predation throughout its lifespan, then continuous mesopredator control may be needed to provide the greatest benefit. Prey species that are both vulnerable throughout their life and which are preyed on by multiple predator species [46], [51], some of which may not be able to be effectively or legally controlled, may not benefit from mesopredator management at all.

For prey species such as birds [10], [13] and ungulates [36] whose vulnerability to predation varies during their annual or life cycle (e.g., nesting season or fawning season), recurring mesopredator removal prior to and during periods of heightened prey vulnerability may reduce mesopredator influence on the prey population. In these cases, continuous control efforts may not be needed; rather, maintaining control efforts only until prey reach a less vulnerable life stage may prove effective. However, some prey may benefit from continuous mesopredator control even when prey are only temporally vulnerable.

Indirect effects of predators may have greater impacts on prey than direct predation [52], [53]. Song sparrow (*Melospiza melodia*) population response to predation risk was greater than the effects of direct predation [53]. Similarly, eastern chipmunks (*Tamias striatus*) altered foraging behavior in response to tufted-titmouse (*Baeolophus bicolor*) alarm calls [52]. Sheriff et al. [54] observed that predation risk increased stress levels and decreased reproduction in snowshoe hares (*Lepus americanus*). Thus, when predators cause prey to alter their behavior such that prey population dynamics are affected, continuous predator control may be needed to bring about a desired prey response.

We had insufficient data to evaluate impacts of removals on mesopredators that may have existed in a metapopulation. If a local population of mesopredators is suspected to exist as part of a metapopulation, special consideration should be given when implanting lethal control. In these cases, mesopredator control can lead to localized extinctions [34]. For example, Beasley et al. [15] trapped raccoons from within forested fragments in an agriculture-dominated landscape; after 3 years, only 40% of fragments had recovered to pre-harvest levels. When dealing with abundant mesopredators existing within metapopulations, localized extinctions or drastically suppressed local populations, as in Beasley et al. [15], may be desirable because of the diminished need for sustained or recurrent removals.

Although not observed in our study, we caution that control efforts carried out over large geographical areas (relative to the target species’ dispersal ability and perhaps much larger than the study areas examined in our study) are more likely to cause longer-lasting population suppression with cumulative negative effects on capture rates occurring with repeated removal efforts. If mesopredator control measures were applied over sufficiently large areas, control would more closely approximate the large spatial scale of harvest typically associated with historical periods of great fur demand which has been suggested to have led to long-term impacts on predator communities [16]. Such long-term population suppression may be viewed negatively with regard to native mesopredators [8], but may be desirable when dealing with invasive predators where complete eradication may be justified [55].

Large carnivores are declining worldwide [1]; whereas, mesopredator populations are generally increasing [3]. In some cases, mesopredator population increases may be the direct result of the loss of large carnivores [56], [57]. Large carnivores are clearly important and their loss carries a great ecological price; however, some ecological costs associated with large carnivore declines may be mitigated by actively managing mesopredator populations. When control of mesopredators is warranted, our analyses suggest that there should be little concern that control of medium and large mesopredators, as typically implemented, will have lasting impacts on their populations. If prey populations benefit from mesopredator removal, our analyses provide evidence that mesopredator control will need to be continuous or recurring for prey populations to continue to benefit. In contrast, where control of small mesopredators may be warranted, control efforts should be coupled with monitoring of small mesopredator abundance. Monitoring results should then be used to determine when control efforts are needed to avoid overharvest and costs associated with unnecessary control efforts.

Mesopredators play an important role in ecosystems, and mesopredator control may have unintended consequences. For example, coyotes, a mesopredator by the definition used herein [2], may enhance prey diversity by suppressing prey populations that possess a competitive advantage over other species [35]. Moreover, some mesopredators may potentially fill roles of extirpated top carnivores [20], [58–60]. This is particularly evident for coyotes [20], [60] and dingoes [59], [61] which have been suggested to suppress smaller mesopredators. However, the conclusion that some mesopredators suppress smaller mesopredators may be premature [62–64] and warrants further study. If some mesopredators act in ways that enhance biodiversity, lethal control may exacerbate, rather than mitigate, undesirable cascading effects. Therefore, lethal control should be applied judiciously and with vigilance toward detecting undesirable consequences such as removal of top-down controls on pest species [20], [60]. Our synthesis should aid in effective application of meospredator control to reduce some, but likely not all, aspects of trophic cascades caused by loss of top carnivores.

In summary, with the exception of small mesopredators, there was little change in removal rate over time in response to mesopredator removal efforts. We suggest that considering mesopredator control efforts as a spatially structured harvest provides a compelling explanation for those mesopredator control efforts that fail to produce a desired prey response (i.e., immigration keeps pace with removals [17]) while at the same time providing a theoretical basis for evaluating potential impacts of mesopredator removals on mesopredator populations. If mesopredators are continuously distributed and the harvested proportion of the landscape remains below that producing a maximum sustained yield, mesopredator populations should not experience long-term suppression as a result of lethal control efforts [34]. Our analyses suggest that this is commonly the case, as mesopredator populations, with the exception of the smallest mesopredators, generally returned to pre-harvest levels within a year.

Because our conclusions are based on analyses of published data, incorporation of additional studies may alter our general findings. However, we suggest this is unlikely. Rather, we predict inclusion of additional data will further confirm our conclusions. We encourage scientists to minimally report number of animals removed, removal effort, and predator population indices or abundance estimates when conducting mesopredator removal studies, as these data are important for continued evaluation of impact of removals on mesopredator populations.

## Supporting Information

S1 Appendix Data used in analyses. (XLSX)

S1 Table A summary of the mesopredator control studies. (DOCX)
