# Supplementary material for: Impacts of Mesopredator Control on Conservation of Mesopredators and Their Prey
Source: PLoS One. 2015 Sep 11;10(9):e0137169. doi: 10.1371/journal.pone.0137169 (PMC4567327; doi:10.1371/journal.pone.0137169)
Supplement: S1 Table — (DOCX) [file pone.0137169.s002.docx]

**S1 Table:** Predator control studies used in these analyses. Grouped references indicate papers involving the same removal effort. Bolded references within grouped references indicate the specific paper used to provide the numbers for the analyses.

| Reference | Species removed^1^ | No. sites with removals | No. years with removals | No. λ values generated^2^ | Index of predator abundance^3^ | Index-suggested effect of removal on predator populations |
| --- | --- | --- | --- | --- | --- | --- |
| Ahola et al. 2006, Banks et al. 2004, Nordström et al. 2002, Nordström et al. 2003, Nordström et al. 2004 | *Neovison vison* | 1 | 9 | 8 | No index |  |
| Balser et al. 1968 | *Procyon lotor, Mephitis mephitis, Vulpes vulpes* | 2 | 3 | 3 | No index |  |
| Bartman et al. 1992 | *Canis latrans* | 1 | 3 | 2 | No index |  |
| Beasom 1974a, Beasom 1974b | *C. latrans, Lynx rufus* | 1 | 2 | 1 | Track counts | Short-term reduction |
| Blackwell et al. 2003 | *Mustela nivalis, Mustela furo, Felis catus* | 1 | 4 | 3 | No index |  |
| Butchko 1990, Cypher & Scrivner 1992 | *C. latrans* | 2 | 6 | 5 | Scent stations | Long-term reduction |
| Chesness et al. 1968 | *M. mephitis, Spilogale putorius, P. lotor, Taxidea taxus, N. vison, Mustela erminea, Mustela frenata, Urocyon cinereoargentus, Didelphis virginiana, F. catus* | 1 | 3 | 2 | No index |  |
| Cuthbert 2002 | *M. erminea* | 1 | 2 | 1 | No index |  |
| Dilks et al. 1996, O’Donnell et al. 1996 | *M. erminea* | 2 | 4 | 5 | No index |  |
| Edminister 1939 | *V. vulpes, U. cinereoargenteus, M. mephitis, P. lotor, F. catus, M. erminea, M. frenata* | 1 | 2 | 1 | No index |  |
| Ellis-Felege et al. 2012 | *P. lotor, D. virginiana, Dasypus novemcinctus, L. rufus, V. vulpes, U. cinereoargenteus,* feral animals | 4 | 6 | 8^4^ | Scent stations | Short-term reduction |
| Fletcher et al. 2010, Fletcher et al. 2013 | *V. vulpes, M. erminea, M. nivalis* | 3 | 7 | 12 | Scat surveys and track tunnels | No effect |
| Frey et al. 2003 | *V. vulpes, C. latrans, P. lotor, M. mephitis, T. taxus, N. vison* | 2^5^ | 3 | 4 | Track counts | No effect |
| Frey and Conover 2007 | *P. lotor, M. mephitis, V. vulpes, N. vison, F. catus, T. taxus* | 1 | 2 | 1 | Spotlight surveys | Short-term reduction |
| Garrettson et al. 1996, Dion et al. 1999, Garrettson and Rohwer 2001, Dion et al. 2003 | *P. lotor, M. mephitis, V. vulpes, T. taxus, N. vison* | 1 | 2 | 1 | Track counts | Short-term reduction |
| Guthery and Beasom 1977 | *C. latrans, M. mephitis, L. rufus, P. lotor, T. taxus, U. cinereoargenteus, D. virginiana* | 1 | 2 | 1 | No index |  |
| Harding et al. 2001 | *V. vulpes* | 1 | 5 | 4 | No index |  |
| Harrington and Conover 2007 | *C. latrans* | 5 | 2 | 5 | No index |  |
| Henke and Bryant 1999 | *C. latrans* | 2^6^ | 2 | 1 | Spotlight surveys | Long-term reduction |
| Hurley et al. 2011 | *C. latrans*^7^ | 4 | 6 | 20 | Scat surveys | No effect |
| Kauhala et al. 1999, Kauhala et al. 2000, Kauhala 2004 | *V. vulpes, Martes martes, N. vison, Nyctereutes procyonoides* | 3 | 5 | 10 | Track counts | Mixed effects |
| Kilgo et al. 2014 | *C. latrans* | 3 | 3 | 6 | Scat surveys | Reduction^8^ |
| Kirkwood et al. 2014 | *V. vulpes* | 1 | 58 | 57 | MNKA | Long-term reduction |
| Korpimäki et al. 2002, Norrdahl et al. 2004, Korpimäki et al. 2005 | *M. nivalis, M. erminea* | 4 | 3 | 8 | Track counts | Mixed effects |
| Lawrence and Silvy 1995 | *M. mephitis, D. virginiana, P. lotor* | 1 | 2 | 1 | No index |  |
| Little and Crowe 2004 | *Cynictis penicillata, Herpestes pulverulentus, Ictonyx striatus, Suricata suricatta, Felis nigripes, Genetta genetta* | 2 | 3 | 3 | No index |  |
| Marcström et al. 1988 | *V. vulpes, M. martes* | 2 | 5 | 7 | No index |  |
| Newsome et al. 1989, Pech et al. 1992 | *V. vulpes*^9^ | 1 | 2 | 1 | Spotlight surveys | Short-term reduction |
| Palmer et al. 2005 | *P. lotor, D. virginiana, U. cinereoargentus, V. vulpes* | 1 | 3 | 2 | Track stations | Short-term reduction |
| Pearson and Caroline 1981^10^ | *C. latrans, L. rufus* | 1 | 4 | 3 | No index |  |
| Pieron and Rohwer 2010, Pieron et al. 2013 | *V. vulpes, C. latrans, P. lotor, T. taxus, M. mephitis, N. vison, M. erminea, M. frenata* | 6 | 3 | 11 | No index |  |
| Steen and Haugvold 2009 | *V. vulpes, M. martes, M. erminea, N. vison* | 1 | 10 | 9 | No index |  |
| Stout 1982 | *C. latrans* | 3 | 4 | 6 | No index |  |
| Summers et al. 2004 | *V. vulpes* | 1 | 5 | 4 | Scat surveys | No effect |

^1^ Removals of species other than mesomammalian predators were not included. ^2^ Not all study sites had removals carried out during all years of the study. ^3^ Populations indices based on the removal efforts themselves are not included. ^4^ One λ value was deemed an outlier and removed from modeling efforts. ^5^ Only Site 2 plot removal numbers were provided; Site 1 numbers not provided by plot and year. ^6^ Annual removal numbers were not given separately by site so sites were lumped together. ^7^ Mountain lions (*Puma concolor*) were also removed. ^8^ Index data not sufficient to determine whether reduction was short of long-term ^9^ *F. catus* also removed, but numbers were not reported by site and year. ^10^ Study reported removals by counties grouped by sheep density. We used only one group of adjacent counties. Removal reports for other groups of counties included counties which were not adjacent and therefore not included here.

**Literature cited**

Ahola M, Nordström M, Banks PB, Laanetu N, Korpimäki E. 2006. Alien mink predation induces prolonged declines in

archipelago amphibians. Proceedings of the Royal Society B. **273**:1261-1265.

Balser DS, Dill HH, Nelson HK. 1968. Effect of predator reduction of waterfowl nesting success. Journal of Wildlife

Management **32**:669-682.

Banks PB, Norrdahl K, Nordström M, Korpimäki E. 2004. Dynamic impacts of feral mink predator on vole metapopulations in

the outer archipelago of the Baltic Sea. OIKOS **105**:79-88.

Bartman RM, White GC, Carpenter LH. 1992. Compensatory mortality in a Colorado mule deer population. Wildlife Monographs

**121**: 2-39.

Beasom, SL. 1974a. Relationships between predator removal and white-tailed deer net productivity. Journal of Wildlife

Management **38**:854-859.

Beasom, SL. 1974b. Selectivity of predator control techniques in south Texas. Journal of Wildlife Management **38**:837-844.

Blackwell GL, Potter MA, McLennan JA, Minot EO. 2003. The role of predators in ship rat and house mouse population

eruptions: drivers or passengers? OIKOS **100**:601-613.

Butchko, PH. 1990. Predator control for the protection of endangered species in California. Proceedings of the Vertebrate Pest

Conference **14**:237-240.

Chesness RA, Nelson MM, Longley WH. 1968. The effect of predator removal on pheasant reproductive success. Journal of

Wildlife Management **32**:683-697.

Cuthbert, R. 2002. The role of introduced mammals and inverse density-dependent predation in the conservation of Hutton’s

shearwater. Biological Conservation **108**:69-78.

Cypher BL, Scrivner JH. 1992. Coyote control to protect endangered San Joaquin kit foxes at the naval petroleum reserves,

California. Proceedings of the Vertebrate Pest Conference **15**:42-47.

Dilks PJ, O’Donnell CFJ, Elliott GP, Phillipson SM. 1996. The effect of bait type, tunnel design, and trap position on stoat

control operations for conservation management. New Zealand Journal of Zoology **23**:295-306.

Dion N, Hobson KA, Lariviѐre S. 1999. Effects of removing duck-nest predators on nesting success of grassland songbirds.

Canadian Journal of Zoology **77**:1801-1806.

Dion N, Hobson KA, Lariviѐre S. 2003. Does removal of duck nest predators affect the temporal patterns of predation for

simulated nests of grassland songbirds? Canadian Field Naturalist **117**:347-351.

Edminister, FC. 1939. The effect of predator control on ruffed grouse populations in New York. Journal of Wildlife Management **3**:345-352.

Ellis-Felege SN, Conroy MJ, Palmer WE, Carroll JP. 2012. Predator reduction results in compensatory shifts in losses of avian

ground nests. Journal of Applied Ecology **49**:661-669.

Fletcher K, Aebischer NJ, Baines D, Foster R, Hoodless AN. 2010. Changes in breeding success and abundance of ground-nesting moorland birds in relation to the experimental deployment of legal predator control. Journal of Applied Ecology **47**:263-272.

Fletcher K, Hoodless AN, Baines D. 2013. Impacts of predator abundance on red grouse *Lagopus lagopus scotica* during a period

of experimental predator control. Wildlife Biology **19**:248-256.

Frey SN, Majors S, Conover MR, Messmer TA, Mitchell DL. 2003. Effect of predator control on ring-necked pheasant

populations. Wildlife Society Bulletin **31**:727-735.

Frey SJ, Conover MR. 2007. Influence of population reduction on predator home range size and spatial overlap. Journal of

Wildlife Management **71**:303-309.

Garrettson PR, Rohwer FC. 2001. Effects of mammalian predator removal on production of upland-nesting ducks in North

Dakota. Journal of Wildlife Management **65**:398-405.

Garrettson PR, Rohwer FC, Zimmer JM, Mense BJ, Dion N. 1996. Effects of mammalian predator removal on waterfowl and

non-game birds in North Dakota. Transactions of the North American Wildlife and Natural Resources Conference **61**:94-101.

Guthery FS, Beasom SL. 1977. Responses of game and nongame wildlife to predator control in south Texas. Journal of Range Management **30**:404-409.

Harding EK, Doak DF, Albertson JD. 2001. Evaluating the effectiveness of predator control: the non-native red fox as a case

study. Conservation Biology **15**:1114-1122.

Harrington JL, Conover MR. 2007. Does removing coyotes for livestock protection benefit free-ranging ungulates? Journal of

Wildlife Management **71**:1555-1560.

Henke SE, Bryant FC. 1999. Effects of coyote removal on the faunal community in western Texas. Journal of Wildlife Management **63**:1066-1081.

Hurley MA, Unsworth JW, Zager P, Hebblewhite M, Garton EO, Montgomery DM, Skalski JR, Maycock CL. 2011. Demographic response of mule deer to experimental reduction of coyotes and mountain lions in southeastern Idaho. Wildlife Monographs **178**:1-33.

Kauhala, K. 2004. Removal of medium-sized predators and the breeding success of ducks in Finland. Folia Zoologica **53**:367-378.

Kauhala, K, Helle P, Helle E. 2000. Predator control and the density and reproductive success of grouse populations in Finland.

Ecography **23**:161-168.

Kauhala K, Helle P, Helle E, Korhonen J. 1999. Impact of predator removal on predator and mountain hair populations in

Finland. Annales Zoologici Fennici **36:**139-148.

Kilgo JC, Vukovich M, Ray HS, Shaw CE, Ruth C. 2014. Coyote removal, understory cover, and survival of white-tailed deer

neonates. Journal of Wildlife Management **78**:1261-1271.

Kirkwood R, Sutherland DR, Murphy S, Dann P. 2014. Lessons from long-term predator control: a case study with the red fox.

Wildlife Research **41**:222-232.

Korpimäki E, Norrdahl K, Klemola T, Pettersen T, Stenseth NC. 2002 Dynamic effects of predators on cyclic voles: field

experimentation and model extrapolation. Proceedings of the Royal Society of London B: Biological Sciences **269**:991-997.

Korpimäki E, Norrdahl K, Huitu O, Klemola T. 2005. Predator-induced synchrony in population oscillations of coexisting

small mammal species. Proceedings of the Royal Society of London B: Biological Sciences **272**:193-202.

Lawrence JS, Silvy NJ. 1995. Effect of predator control on reproductive success and hen survival of Attwater’s Prairie-chicken.

Proceedings of the Annual Association of Fish and Wildlife Agencies **49**:275-282.

Little RM, Crowe TM. 2004. Effects of a predator control experiment on Grey-winged Francolin (*Scleroptila africanus*) populations.

Ostrich **75**:285-287.

Marcström V, Kenward RE, Engren E. 1988. The impact of predation on boreal tetraonids during vole cycles: an experimental

study. Journal of Animal Ecology **57**:589-872.

Newsome AE, Parer I, Catling PC. 1989. Prolonged prey suppression by carnivores: predator-removal experiments. Oecologia **78**:458-467.

Nordström M, Högmander J, Nummelin J, Laine J, Laanetu N, Korpimäki E. 2002. Variable responses of waterfowl breeding

populations to long-term removal of introduced American mink. Ecography **25**:385-394.

Nordström M, Högmander J, Laine J, Nummelin J, Laanetu N, Korpimäki E. 2003. Effects of feral mink removal on seabirds,

waders and passerines on small islands in the Baltic Sea. Biological Consevation **109**:359-368.

Nordström M, Laine J, Ahola M, Korpimäki E. 2004. Reduced nest defense intensity and improved breeding success in terns as

responses to removal of non-native American mink. Behavioral Ecology and Sociobiology **55**:454-460.

Norrhahl K, Heinilä H, Klemola T, Korpimäki E. 2004. Predator-induced changes in population structure and individual quality

of *Microtus* voles: a large-scale field experiement. OIKOS **105**:312-324.

O’Donnell CFJ, Dilks PJ, Elliott GP. 1996. Control of a stoat (*Mustela erminea*) population irruption to enhance mohua

(yellowhead) (*Mohoua ochrocephala*) breeding success in New Zealand. New Zealand Journal of Zoology **23**:279-286.

Palmer WE, Wellendorf SD, Gillis JR, Bromley PT. 2005. Effect of field borders and nest-predator reduction on abundance of northern bobwhite. Wildlife Society Bulletin **33**:1398-1405.

Pearson EW, Caroline M. 1981. Predator control in relation to livestock losses in central Texas. Journal of Range Management **34**:435-441.

Pech RP, Sinclair ARE, Newsome AE, Catling PC. 1992. Limits to predator regulation of rabbits in Australia: evidence from

predator-removal experiments. Oecologia **89**:102-112.

Pieron MR, Rohwer FC. 2010. Effects of large-scale predator reduction on nest success of upland nesting ducks. Journal of Wildlife Management **74**:124-132.

Pieron MR, Rohwer FC, Chamberlain MJ, Kaller MD, Lancaster J. 2013. Response of breeding duck pairs to predator reduction in North Dakota. Journal of Wildlife Management **77**:663-671.

Steen JB, Haugvold OA. 2009. Cause of death in willow ptarmigan *Lagopus l. lagopus* chicks and the effect of intensive, local

predator control on chick production. Wildlife Biology **15**:53-59.

Stout, GG. 1982. Effects of coyote reduction on white-tailed deer productivity on Fort Still, Oklahoma. Wildlife Society Bulletin **10**:329-332.

Summers RW, Green RE, Procter R, Dugan D, Lambie D, Moncrieff R, Moss R, Baines D. 2004. An experimental study of the effects of predation on the breeding productivity of capercaillie and black grouse. Journal of Applied Ecology **41**:513-525.
